# Supplementary material for: The interactome of intact mitochondria by cross-linking mass spectrometry provides evidence for coexisting respiratory supercomplexes
Source: Mol Cell Proteomics. 2017 Dec 8;17(2):216–32. doi: 10.1074/mcp.RA117.000470 (PMC5795388; doi:10.1074/mcp.RA117.000470)
Supplement: Supplemental Data [file supp_RA117.000470_133922_0_supp_23978_fzffwf.zip › spectra_annotation/mito_spectra_annotation/84-1-9-1-5-1.pdf]

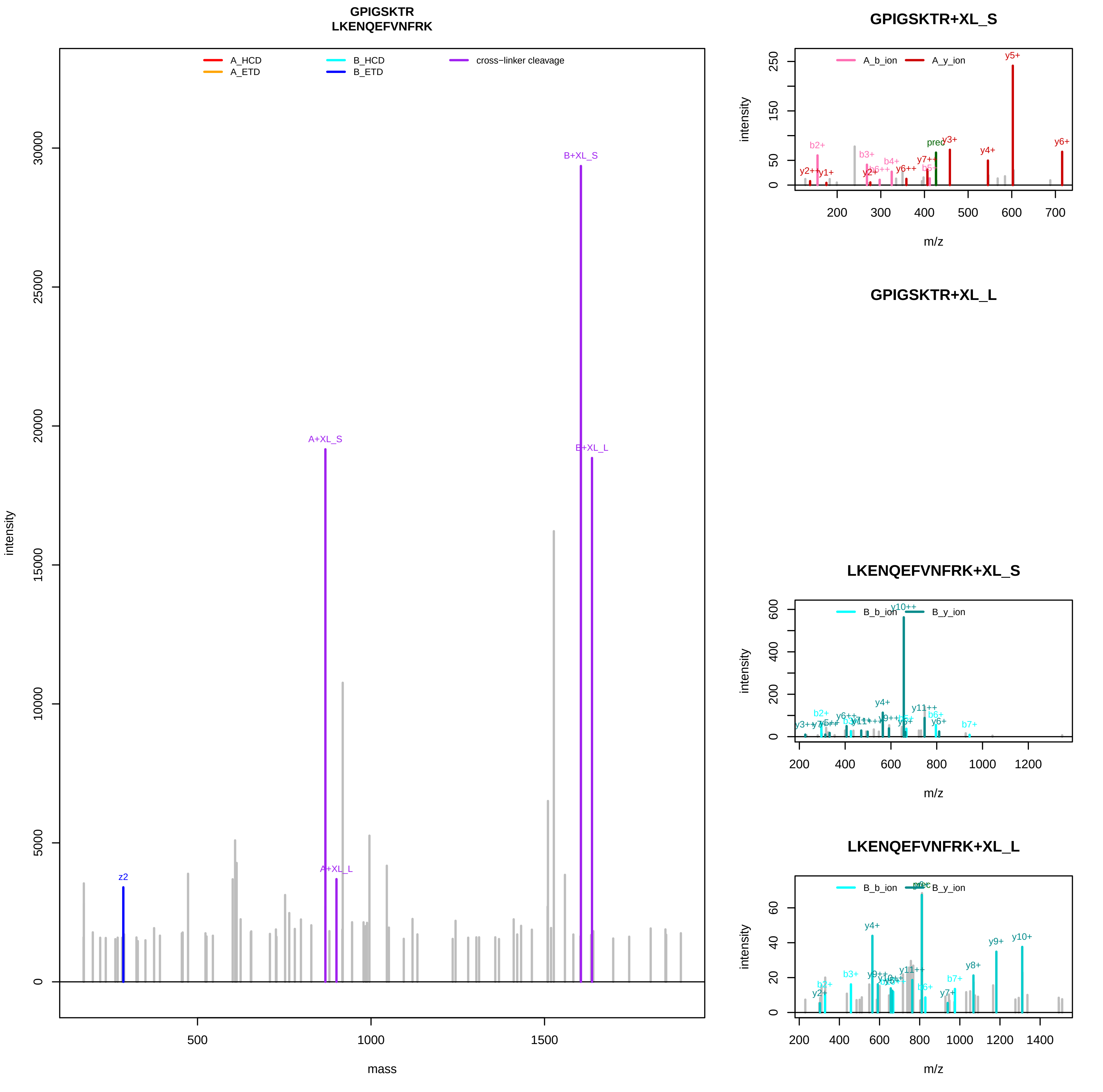

GPIGSKTR
LKENQEFVNFRK
A_HCD
A_ETD
B_HCD
B_ETD
cross−linker cleavage
B+XL_S
A+XL_S
B+XL_L
A+XL_L
z2
intensity
mass
0
5000
10000
15000
20000
25000
30000
500
1000
1500
GPIGSKTR+XL_S
A_b_ion
A_y_ion
y5+
y6+
y3+
y4+
prec
b2+
b3+
b4+
y2++
y1+
y2+
y6++
y7++
b5+
m/z
GPIGSKTR+XL_L
LKENQEFVNFRK+XL_S
B_b_ion
B_y_ion
y10++
y4+
y11++
b2+
b6+
y6+
b7+
LKENQEFVNFRK+XL_L
prec
y4+
b3+
y9+
y10+
y8+
b7+
y7+
b2+
y2+
y11++
